# Short-term HRV metrics as a method for intraoperative assessment of cardiac parasympathetic response to rapid atrial pacing

**DOI:** 10.3389/fphys.2026.1753958

**Published:** 2026-02-26

**Authors:** Przemysław Skoczyński, Bruno Hrymniak, Bartosz Skonieczny, Krystian Josiak, Bartosz Biel, Antoni Wileczek, Katarzyna Czarnecka, Sebastian Stec, Waldemar Banasiak, Dorota Zyśko, Dariusz Jagielski

**Affiliations:** 1 Department of Cardiology, Center for Heart Diseases, 4th Military Clinical Hospital, Wroclaw, Poland; 2 Department of Emergency Medicine, Wroclaw Medical University, Wroclaw, Poland; 3 Faculty of Medicine, Wrocław University of Science and Technology, Wroclaw, Poland; 4 Panaceum-Med, Sanok, Poland; 5 American Heart of Poland, Dąbrowa Górnicza, Poland; 6 Department of Cardiac Surgery and Transplantation, National Medical Institute of the Ministry of the Interior and Administration, Warsaw, Poland; 7 ELMedica, EP-Network, Skarżysko-Kamienna, Poland

**Keywords:** autonomic nervous system, cardioneuroablation (CNA), electrophysiological study (EPS), HRV (heart rate variability), parasympathetic nervous system

## Abstract

**Introduction:**

Heart rate variability (HRV) is widely used to assess parasympathetic influence on cardiac function and has proven useful in evaluating long-term autonomic effects of cardioneuroablation (CNA). However, HRV has not yet been used intraoperatively to quantify dynamic, short-term changes in parasympathetic tone. Rapid atrial pacing (AP) is expected to provoke a brief parasympathetic reaction, but no standardized method exists to assess this response in real time during electrophysiological procedures.

**Aims:**

To evaluate HRV changes induced by rapid AP using RMSSD and the maximal-minimal PP interval difference (ΔPP), and to assess the feasibility of repeated intraoperative monitoring.

**Methods:**

This prospective observational study enrolled 50 patients (median age 39 years [IQR 31-52]) without structural heart disease referred for electrophysiological study. RMSSD and ΔPP were calculated from four PP intervals before pacing and reassessed immediately after 30-s atrial pacing at 100 bpm. Heart rate, Sinus node recovery time, cSNRT and Wenckebach point were also measured. All measurements were repeated 2 minutes later.

**Results:**

Rapid AP produced a significant increase in RMSSD (15.7 ms [9.7–23.7] vs. 41.7 ms [25.6–59.6], p < 0.001) and ΔPP (33 ms [19-56] vs. 90 ms [60-152], p < 0.001). The response was reproducible in the second pacing sequence (RMSSD 13.6→41.0 ms; ΔPP 24→107 ms; both p < 0.001; Wilcoxon signed-rank test with Bonferroni correction). HRV changes occurred independently of sinus cycle length modifications. No significant differences were observed in SNRT, cSNRT, or Wenckebach point.

**Conclusion:**

Rapid AP evokes a robust, repeatable parasympathetic response detectable using ultra-short HRV metrics-expressed as an increase in RMSSD and ΔPP. These parameters allow real-time intraoperative assessment of parasympathetic influence on the sinus node. This approach warrants validation in future studies involving CNA, atropine challenge, and ECVS.

## Highlights

- Rapid atrial pacing induces a measurable parasympathetic response, expressed as increases in RMSSD and ΔPP. These heart rate variability parameters can be assessed intraoperatively, offering a simple and repeatable method to quantify vagal modulation of the sinus node during electrophysiological procedures. The reproducibility of this response within a single intervention supports its potential utility for monitoring autonomic effects in real time. This approach may be particularly valuable for evaluating the impact of ablation strategies such as pulmonary vein isolation or cardioneuroablation. However, it reflects sinus node activity only and requires further validation.

## Introduction

In recent years, the repertoire of electrophysiological manoeuvres has expanded to include extracardiac vagal nerve stimulation (ECVS), which enables controlled induction of marked hypervagotonia. This phenomenon has proven valuable in assessing parasympathetic influence on the cardiac pacemaking and conduction system, as well as its modulation. It is now used intraoperatively to evaluate the effectiveness of cardioneuroablation (CNA) (Pachon et al., 2015).

CNA, as a method of modulating the parasympathetic influence on the heart’s pacemaking and conduction system, is finding increasing use in the treatment of reflex syncope in vasovagal syndrome (VVS) as well as in functional, symptomatic bradycardia resulting from both sinus node dysfunction (SND) and various degrees of atrioventricular blocks (AVB) (Pachon et al., 2005; Pachon et al., 2011; Piotrowski et al., 2022; Aksu et al., 2021), which -until recently - were considered irreversible and indicated implantation of a pacemaker (PM) (Glikson et al., 2021). The possibility of modifying parasympathetic tone requires intraoperative assessment. The ECVS technique introduced by Pachon requires general anesthesia and patient paralysis, which significantly complicates its use and limits its availability (Pachon et al., 2015). Despite its limitations and the lack of a standardized methodology for performing CNA, ECVS is generally regarded as the reference method. It is commonly employed to assess the intra-procedural endpoint of CNA both in the treatment of cardioinhibitory VVS and in functional bradycardia (Piotrowski et al., 2022; Reichert et al., 2022; Stodółkiewicz-Nowarska et al., 2024; Skoczyński et al., 2024). The atropine test, which is also effective in assessing vagal influence on the heart, has the significant limitation of atropine’s prolonged action, which prevents repeated use during a single procedure (Pachon et al., 2005; Aksu et al., 2019). Non-invasive assessment of parasympathetic tone using heart rate variability (HRV) analysis has been used for many years but is based on long-term Holter monitoring. Its usefulness in evaluating long-term CNA effects has been demonstrated (Pachon et al., 2020); however, it has not yet been used for intraoperative assessment of parasympathetic tone. The only publication attempting to use HRV assessment during EPS indicates its usefulness in assessing sympathetic responses (Fuenmayor et al., 1996).

The sympathetic-parasympathetic balance is a dynamic state, modified by many factors, including heart rate and cardiac output. It has been hypothesized that both tachyarrhythmias and imposed rapid AP potentially associated with transient changes in cardiac output may evoke compensatory parasympathetic modulation via baroreceptor engagement (La Rovere et al., 1995). Until now, however, HRV analysis has not been used intraoperatively to assess parasympathetic tone and its changes under the stimulus of rapid pacing. The possibility of intraoperative assessment of the short-term parasympathetic response to rapid atrial pacing (AP) could enable multiple, repeatable measurements. In the future, this could allow its use as a method for intraoperative evaluation of the effectiveness of procedures that modulate parasympathetic tone, such as CNA.

### Aims

- To evaluate changes in HRV in response to rapid AP, based on the variation in RMSSD and ΔPP (difference between the longest and shortest PP intervals), measured before and after atrial stimulation.
- To assess the feasibility of intra-procedural, repeatable monitoring of HRV response to rapid AP, using changes in RMSSD and ΔPP, measured in pre- and post-stimulation phases.

## Materials and methods

This was a prospective, single-center observational study involving 50 patients (median age 39 years [IQR 31-52]; 46% male) referred for invasive electrophysiological study (EPS) due to palpitations. All patients had no comorbidities, structurally normal hearts, and no evidence of sinus node dysfunction (SND) or atrioventricular conduction abnormalities. Inducible arrhythmias were not identified during EPS. Both diseases and pharmacotherapy that could affect the autonomic nervous system were criteria for exclusion from the study. Patients were not treated with beta-blockers, antiarrhythmic medications, or ivabradine. On the day of the study, they were fasting. The procedure was performed under local anesthesia in the area of vascular access, and no sedative or calming medications were used. The baseline demographic characteristics of the cohort are presented in Table 1. All patients provided written informed consent to participate in the study, which was approved by the Bioethics Committee of the Wroclaw Medical University (KB 622/2024).

**TABLE 1:** Demographic characteristics.

Age, years, median (IQR)	39 (31–52)
Male gender, n (%)	23 (46)
Weight (kg) median (IQR)	72.5 (60–88)
Height (cm), median (IQR)	171 (165–183)

### Electrophysiological protocol

The study protocol was performed during standard EPS. Each subject underwent 2 standardized pacing protocols, applied before (first protocol) and 2 min after (second protocol) the diagnostic EPS.

Each protocol consisted of:

- resting measurement of spontaneous sinus rhythm, labeled as period A (first protocol) and period C (second protocol) – 4 cycles before rapid AP
- a 30-s AP at 100 bpm, followed by a second measurement, labeled as period B and period D, respectively.

**FIGURE 1:**
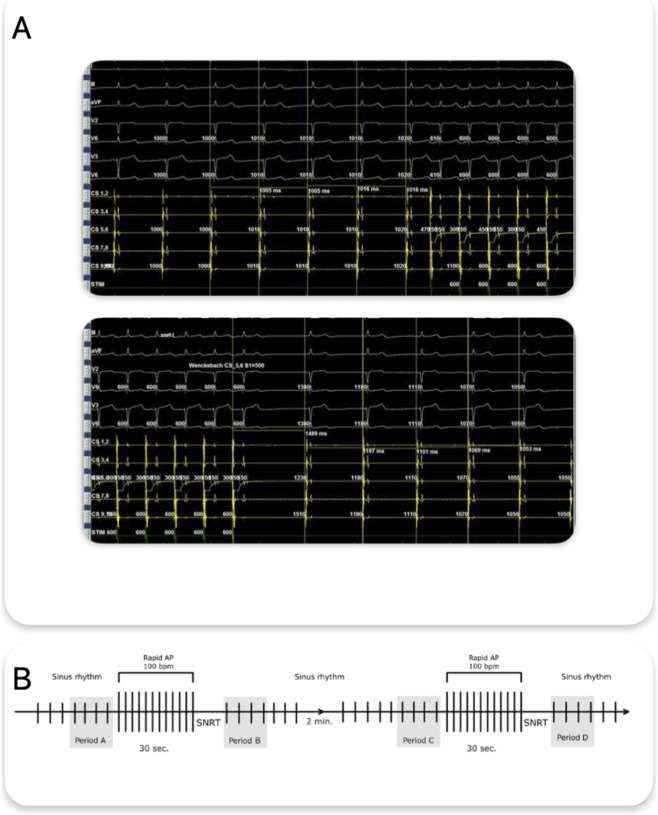
**(A)** Methodology of PP cycle measurement - four consecutive PP intervals preceding atrial rapid pacing and the first four PP intervals after the return of sinus rhythm following pacing termination are shown. Measurements were obtained from recordings of the coronary sinus electrogram. **(B)** Schematic representation of the protocol and predefined analysis periods (A-D). AP, Atrial Pacing, SNRT, Sinus Node Recovery Time.

Just before stimulation, 4 consecutive sinus cycles (PP intervals) were manually measured from high-resolution intracardiac tracings - period A and C and the first 4 cycles after the return of sinus rhythm following pacing (period B and period D) (Figures 1A,B).

Both PP interval measurements and atrial stimulation were performed using a ten-pole electrode placed in the coronary sinus. Measurements and stimulation were always performed from the same electrode rings for each patient. If a measurement was contaminated by extrasystole, artifacts, or ineffective stimulation, it was not considered and was repeated after 2 min.

From these 4 cycles, the following time-domain HRV parameters were calculated:

- ΔPP (in milliseconds), defined as the difference between the longest and shortest PP intervals;
- RMSSD (root mean square of successive differences), calculated as:

$$RMSSD=\sqrt{\frac{\sum_{i=1}^{n-1}{\left({PP}_{i+1}-{PP}_{i}\right)}^{2}}{n-1}}$$

where n = 4 (Shaffer et al., 2014).

These indices were chosen to estimate short-term vagal modulation of sinus node activity in response to AP. Both metrics reflect beat-to-beat variability and are physiologically sensitive to parasympathetic tone.

In addition, standard electrophysiological parameters were recorded:

- Sinus node recovery time (SNRT) and its corrected form (cSNRT, defined as SNRT - baseline cycle length),
- Atrio-ventricular Wenckebach point (WP), defined as the highest AP rate with 1:1 AV conduction.

All measurements were taken in sinus rhythm under resting, non-sedated conditions, using standard quadripolar catheters and a multi-channel recording system.

### Statistical analysis

Short-term sinus rhythm variability was assessed using 2 time-domain indices derived from 4 consecutive PP intervals during predefined periods (labeled A-D):

- ΔPP, defined as the difference between duration of the longest and shortest PP interval within the 4-cycle window (PP_max - PP_min);
- RMSSD.

Variables are presented as medians and IQR. Normality was assessed using the Shapiro-Wilk test. Correlations between paired measurements were evaluated using Spearman’s rank correlation coefficient (ρ) when normality assumptions were not met. Differences across repeated measures (periods A-D) were analyzed using Friedman’s non-parametric ANOVA. Post-hoc testing focused on the prespecified pacing contrasts (A vs. B and C vs. D) and was performed using two-sided Wilcoxon signed-rank tests with Bonferroni correction for two comparisons (significance threshold p < 0.025). Exact p-values are reported where possible; very small values are reported as p < 0.001.

To assess measurement stability and the effect of pacing on autonomic tone, the following steps were undertaken:

- Bland-Altman analysis was used to evaluate agreement between paired conditions and to quantify changes induced by atrial pacing. For each comparison, we calculated:
- Bias (mean difference),
- Standard deviation (SD) of the differences,
- Relative bias (%bias) - bias divided by the mean of the pair.

For Bland-Altman comparisons, differences were defined as the first period minus the second period (e.g., A−B, C−D). Therefore, negative bias indicates higher values in the second (post-pacing) period.

- Second-order difference plots (SODP) were used as an exploratory visualization. For each subject and metric, we computed consecutive between-period differences across A-D (Δ1 = B-A, Δ2 = C-B, Δ3 = D-C) and plotted (Δ1,Δ2) and (Δ2,Δ3). SODP was used to highlight directional consistency across epochs and to flag potential outliers for cautious interpretation.

Given the short sampling window (4 cycles per measurement), limits of agreement (LoA) were not reported, as their interpretation would be statistically unstable and potentially misleading in this context. Instead, bias and %bias were prioritized as the main descriptors of agreement and effect size for both RMSSD and ΔPP.

To explore the comparative sensitivity of RMSSD and ΔPP in detecting pacing-induced autonomic changes, we analyzed the frequency of positive deltas (post-pre stimulation) for each index during the first (A→B) and second (C→D) pacing protocols. A positive delta was defined as any increase in the respective metric following atrial pacing (Table 2).

**TABLE 2:** ΔPP and RMSSD pacing-induced changes.

Comparison	Metric	Patients with Δ > 0 (n)	Patients with Δ > 0 (%)
A vs. B	ΔPP	43	86
A vs. B	RMSSD	41	82
C vs. D	ΔPP	47	94
C vs. D	RMSSD	43	86

All analyses were performed using DataTab (https://datatab.net). A p-value below 0.05 was considered statistically significant.

## Results

**FIGURE 2:**
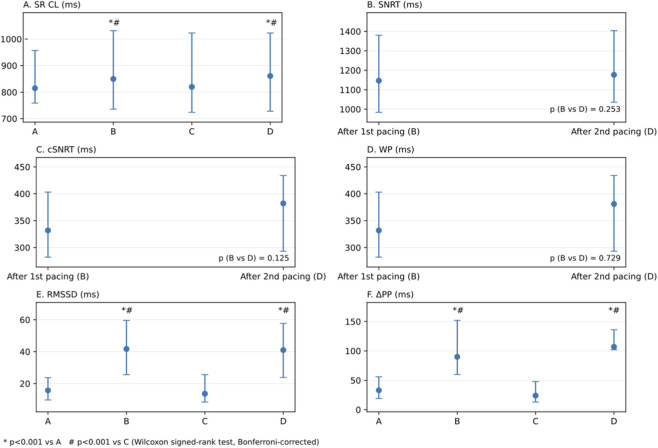
Median (IQR) values across periods **(A–D)** for **(A)** sinus rhythm cycle length (SR CL), **(B)** sinus node recovery time (SNRT), **(C)** corrected SNRT (cSNRT), **(D)** Wenckebach point (WP), **(E)** RMSSD, and **(F)** ΔPP. SNRT, cSNRT, and WP are shown for post-pacing periods **(B)** and **(D)**. p < 0.001 vs A; #p < 0.001 vs C (Wilcoxon signed-rank test, Bonferroni-corrected). SRCL, Sinus Rhythm Cycle Length; SNRT, Sinus Node Recovery Time; cSNRT, corrected Sinus Node Recovery Time; WP, Wenckebach Point.

The study confirmed a physiological phenomenon consisting of an increase in HRV and prolongation of the median SRCL in response to rapid AP during EPS. A significant increase in the median sinus rhythm cycle length (SR CL), as well as ΔPP and RMSSD, was observed after rapid AP in both pacing sequences (A vs. B: RMSSD p < 0.001, ΔPP p < 0.001; C vs. D: RMSSD p < 0.001, ΔPP p < 0.001; Wilcoxon signed-rank test with Bonferroni correction). Baseline measurements were comparable (A vs. C: RMSSD p = 0.145; ΔPP p = 0.274) and post-pacing values did not differ between the two pacing sequences (B vs. D: RMSSD p = 0.499; ΔPP p = 0.688). Furthermore, SNRT, cSNRT, and WP did not significantly differ between the two post-pacing measurements (B vs. D: p = 0.253, p = 0.125, and p = 0.729, respectively) (Table 3; Figure 2).

**TABLE 3:** SR CL, SNRT, cSNRT, WP and HRV parameters across stimulation periods. SNRT, cSNRT and Wenckebach point were assessed only after pacing, corresponding to periods B and D.

Parameter	Period	p-value
SR CL before 1^st^ pacing, (ms), median (IQR)	A	815 (759–957)
SR CL after, 1^st^ pacing (ms), median (IQR)	B	850 (736-1032) *#
SR CL before 2^nd^ pacing, (ms), median (IQR)	C	820 (724–1023)
SR CL after, 2^nd^ pacing (ms), median (IQR)	D	861 (728-1023) *#
SNRT after 1st pacing (period B), ms, median (IQR)		1147 (984–1380)
SNRT after 2nd pacing (period D), ms, median (IQR)		1177 (1036–1404)
p (B vs. D)		0.253
cSNRT after 1st pacing (period B), ms, median (IQR)		332 (282–403)
cSNRT after 2nd pacing (period D), ms, median (IQR)		382 (293–434)
p (B vs. D)		0.125
WP after 1st pacing (period B), ms, median (IQR)		332 (282–403)
WP after 2nd pacing (period D), ms, median (IQR)		381 (293–434)
p (B vs. D)		0.729
RMSSD before 1^st^ pacing, (ms), median (IQR)	A	15.7 (9.7–23.7)
RMSSD after, 1^st^ pacing (ms), median (IQR)	B	41.7 (25.6–59.6) *#
RMSSD before 2^nd^ pacing, (ms), median (IQR)	C	13.6 (8.4–25.6)
RMSSD after, 2^nd^ pacing (ms), median (IQR)	D	41.0 (23.8–57.7) *#
ΔPP before 1^st^ pacing, (ms), median (IQR)	A	33 (19–56)
ΔPP after, 1^st^ pacing (ms), median (IQR)	B	90 (60-152) *#
ΔPP before 2^nd^ pacing, (ms), median (IQR)	C	24 (13–48)
ΔPP after, 2^nd^ pacing (ms), median (IQR)	D	107 (102-136) *#

SRCL, Sinus Rhythm cycle length; SNRT, Sinus Node Recovery Time; cSNRT, corrected Sinus Node Recovery Time; WP, Wenckebach point; HRV, Heart Rate Variability.

*- p < 0.001 vs. A (Wilcoxon signed-rank, Bonferroni-corrected).

#- p < 0.001 vs. C (Wilcoxon signed-rank, Bonferroni-corrected).

The Bland-Altman analysis was applied to evaluate changes in short-term HRV - RMSSD and ΔPP - across 4 predefined conditions: baseline before the first stimulation (A), post-first stimulation (B), pre-second stimulation (C), and post-second stimulation (D). The following comparisons were analyzed: A vs. B, A vs. C, C vs. D, and B vs. D. For each pair, the bias, standard deviation (SD) of differences, and relative bias (%) were calculated (Table 4 for ΔPP; Table 5 for RMSSD).

**TABLE 4:** Bland-Altman agreement analysis for ΔPP.

Periods	Bias [ms]	SD [ms]	Bias (%)
A vs. B	−84.25	98.38	−19.31
A vs. C	+4.92	34.65	+13.49
C vs. D	−76.44	68.16	−24.42
B vs. D	+12.73	117.55	+10.15

**TABLE 5:** Bland-Altman agreement analysis for RMSSD.

Periods	Bias [ms]	SD [ms]	Bias (%)
A vs. B	−33.33	54.14	−43.34
A vs. C	+2.88	16.70	+4.90
C vs. D	−29.63	37.58	−38.30
B vs. D	+7.92	59.12	+9.01

**FIGURE 3:**
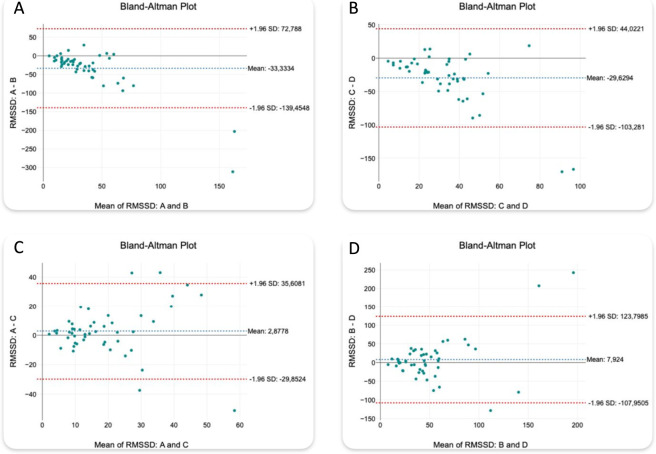
Bland-Altman plots comparing RMSSD across stimulation periods. **(A)** A vs B, **(B)** C vs D, **(C)** A vs C, **(D)** B vs D.

In the comparison of A vs. B (before vs. after the first stimulation), RMSSD increased substantially, reflected by a negative bias (A−B: −33.33 ms; SD: 54.14 ms; Table 5; Figure 3A). A similar pattern was observed for C vs. D (C−D: −29.63 ms; SD: 37.58 ms; Table 5; Figure 3B). The baseline comparison A vs. C showed minimal bias (A−C: +2.88 ms; SD: 16.70 ms; Table 5; Figure 3C), supporting good stability of resting RMSSD. In contrast, the post-pacing comparison B vs. D showed a small positive bias (B−D: +7.92 ms; Table 5; Figure 3D) with the largest dispersion (SD: 59.12 ms), indicating substantial inter-individual variability in response to repeated pacing.

**FIGURE 4:**
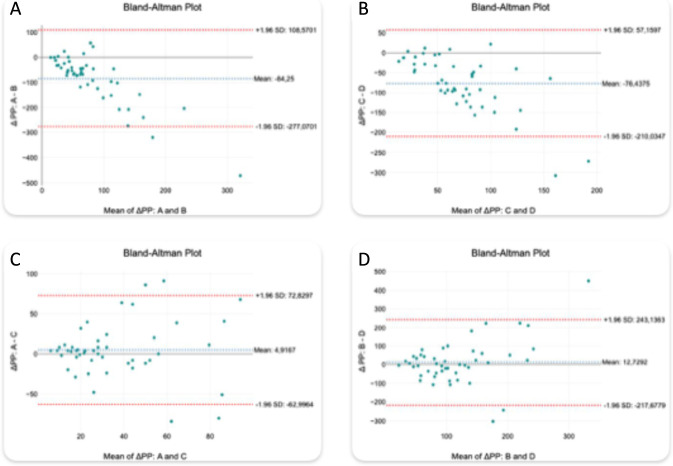
Bland-Altman plots comparing ΔPP across stimulation periods. **(A)** A vs B, **(B)** C vs D, **(C)** A vs C, **(D)** B vs D.

ΔPP showed a complementary pattern. After pacing, ΔPP increased markedly, reflected by negative biases in A vs. B (A−B: −84.25 ms; SD: 98.38 ms; Table 4; Figure 4A) and C vs. D (C−D: −76.44 ms; SD: 68.16 ms; Table 4; Figure 4B). These consistent increases in variability support the hypothesis of a sympathetic-parasympathetic compensatory mechanism. The baseline comparison A vs. C demonstrated only a small bias (A−C: +4.92 ms; SD: 34.65 ms; Table 4; Figure 4C), whereas the post-pacing comparison B vs. D showed a small positive bias (B−D: +12.73 ms; Table 4; Figure 4D) with the largest dispersion (SD: 117.55 ms).

However, the Shapiro-Wilk test indicated non-normality of the differences for both A vs. C (p = 0.0082) and B vs. D (p < 0.001); therefore, Spearman’s rank correlation was used. Agreement was moderate at rest (A vs. C: ρ = 0.553, p < 0.001) and weak post-pacing (B vs. D: ρ = 0.344, p = 0.018).

The Shapiro-Wilk test confirmed non-normality in both A vs. C (p = 0.0021) and B vs. D (p < 0.001) comparisons for ΔPP, and Spearman correlation analysis showed a moderate agreement for A vs. C (ρ = 0.558, p < 0.001) and weak agreement for B vs. D (ρ = 0.271, p = 0.072), indicating that repeatability was preserved at rest but varied substantially post-intervention.

Notably, the Bland-Altman plots (Figures 3, 4) included a small number of strong outliers. As ectopy was carefully excluded during segment selection, these extreme points are more likely related to the high sensitivity of ultra-short (4-cycles) variability estimates to single-beat annotation uncertainty (e.g., subtle P-wave delineation issues, transient noise, or borderline cycle-length marking). Nevertheless, true inter-individual heterogeneity in the autonomic response to rapid AP cannot be excluded; therefore, these observations were interpreted cautiously in the context of the overall group-level pattern.

**FIGURE 5:**
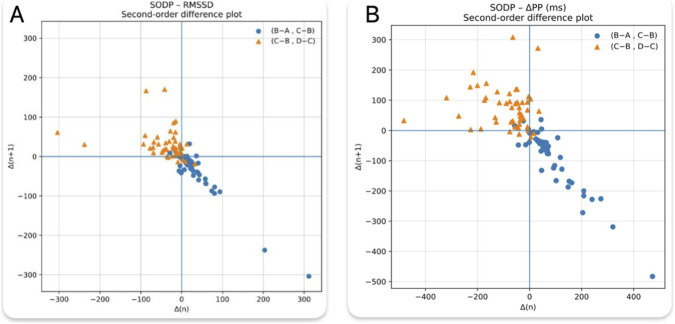
SODP for **(A)** RMSSD and **(B)** ΔPP across periods (A-D). Points represent consecutive between-period differences (B-A, C-B) and (C-B, D-C); axes show Δ(n) and Δ(n+1) (ms).

SODP provided an intuitive visualization of the temporal sequence of changes across the protocol (Figure 5). For both RMSSD-derived and ΔPP measures, most observations mapped to the (+,-) quadrant for (B-A, C-B), indicating an increase immediately after pacing followed by a decrease after the 2-min pause, while the corresponding (C-B, D-C) points predominantly mapped to the (-,+) quadrant, reflecting recovery followed by a renewed increase after the second pacing cycle. SODP additionally facilitated visual identification of these extreme observations, which deviated from the predominant directional pattern.

## Discussion

The main finding of this study is the identification of a distinct physiological response whereby rapid AP transiently increases parasympathetic tone, a reaction that can be reliably assessed intraoperatively during EPS using changes in RMSSD and ΔPP derived from only four consecutive PP intervals. Importantly, this response appears to be independent of respiratory influence.

A significant rise in both RMSSD and ΔPP was observed immediately after pacing. This parasympathetic response proved to be short-lived, as sinus rate, RMSSD, and ΔPP returned to baseline within no more than 2 minutes after pacing cessation.

The mechanism underlying this phenomenon is likely multifactorial. Rapid AP may transiently alter hemodynamics (e.g., cardiac output and arterial pressure), which could shift autonomic balance toward increased vagal modulation and trigger compensatory reflex responses aimed at restoring homeostasis. One plausible contributor is the carotid baroreceptor reflex, whereby a rise in arterial pressure activates stretch receptors and can elicit parasympathetic-mediated bradycardia and a hypotensive response (La Rovere et al., 1995). In our study, the absence of concurrent blood pressure monitoring precludes direct confirmation that this pathway accounted for the observed HRV changes.

Because enhanced vagal activity produces sinus rhythm cycle length changes consistent with those observed in our study, we consider the pacing-induced increase in RMSSD and ΔPP to represent a transient surge in parasympathetic output (DeGiorgio et al., 2010). Importantly, this reaction proved to be reproducible, as an almost identical response was observed during the second pacing maneuver.

Abel et al. also investigated the impact of rapid AP on HRV. However, in contrast to our findings, they reported a decrease in HRV - assessed as ΔRRDEV - accompanied by a rise in heart rate, which they interpreted as a sympathetic response (Fuenmayor et al., 1996). This interpretation was supported by elevated plasma levels of adrenaline and noradrenaline following both atrial and ventricular pacing, indicating sympathetic activation (Hull et al., 1990; Fuenmayor et al., 1992).

Several methodological differences may explain the discrepancy between their results and ours. In Abel’s cohort of 41 patients, only 16 had no prior diagnosis of heart failure; thus, physiological autonomic responses to pacing could be reliably assessed only in this subgroup, whereas autonomic control is known to be altered in heart failure. Moreover, their HRV assessment was based on 30-s segments before and after rapid pacing, substantially longer than in our protocol, which focused on ultra-short, beat-to-beat responses.

These differences suggest that parasympathetic augmentation induced by rapid AP may be a very short-lasting phenomenon, detectable only within the first seconds after pacing, and potentially masked when longer HRV windows are used. A similar pattern is seen in other maneuvers that enhance vagal tone, such as the Valsalva maneuver, where the parasympathetic surge is likewise brief.

Secondly, the Bland-Altman analysis of both RMSSD and ΔPP revealed a consistent post-stimulation increase in vagal tone parameters across the population. However, the wide limits of agreement - particularly in comparisons A vs. B and C vs. D - highlighted marked inter-individual variability in parasympathetic responsiveness. While most patients demonstrated a clear shift toward elevated RMSSD and increased ΔPP after rapid AP, others showed marginal or even paradoxical responses, possibly reflecting variability in intrinsic autonomic tone, compensatory capacity, or psychological stress at baseline.

This heterogeneity suggests that while short-term HRV measures can reliably detect a general group-level trend of vagal reactivation post-pacing, individual responses may differ substantially. Interestingly, the second stimulation series (C vs. D) demonstrated slightly narrower dispersion and fewer extreme negative responders, which may point to habituation, reduced sympathetic arousal, or procedural desensitization during the latter part of the protocol. This reinforces the notion that procedural context and patient-specific autonomic dynamics must be considered when interpreting short-term HRV fluctuations.

Moreover, while both ΔPP and RMSSD showed measurable changes in response to AP, ΔPP more frequently exhibited post-stimulation increases (88% and 98% of patients for A→B and C→D, respectively) than RMSSD (84% and 90%) (Table 2). At first glance, this might suggest that ΔPP is more sensitive in detecting immediate beat-to-beat changes. However, this observation warrants careful interpretation.

Because ΔPP is calculated from the extremes of a small sample of PP intervals, it is particularly vulnerable to the influence of single aberrant values, which can disproportionately affect its magnitude. This can result in exaggerated shifts in response to isolated sinus acceleration or deceleration, even in the absence of consistent autonomic modulation. By contrast, RMSSD reflects the average magnitude of beat-to-beat differences and is less influenced by extreme values, providing a smoother and physiologically integrated measure of vagal tone.

Furthermore, the Bland-Altman analysis revealed substantially higher inter-subject dispersion for ΔPP compared to RMSSD, particularly in the B vs. D comparison (SD 117.6 ms vs. 59.1 ms), suggesting that ΔPP may be more susceptible to inter-individual fluctuations and less reproducible.

Nonetheless, the higher frequency of detected changes supports the potential utility of ΔPP in intra-procedural settings where rapid identification of physiological responses is critical. Its simplicity, rapid calculation, and visual interpretability make it a practical tool, especially when time and beat sampling are limited. However, its lower statistical stability relative to RMSSD highlights the importance of interpreting ΔPP in context and, where possible, corroborating findings using complementary HRV indices.

Third, both RMSSD and ΔPP appear to be largely independent of respiratory sinus arrhythmia. This is supported by the high repeatability of measurements obtained in the two baseline periods (A and C) as well as in the two post-stimulation periods (B and D). In the absence of pacing, RMSSD and ΔPP showed minimal differences between periods A and C, indicating that respiratory variability did not meaningfully influence these ultra-short measurements.

Rapid AP produced a significant and reproducible increase in both RMSSD and ΔPP (periods B and D), confirming that the observed changes reflect pacing-induced autonomic modulation rather than spontaneous respiratory fluctuations. This independence from respiratory variability allows the assessment window for RMSSD and ΔPP to be shortened to only four consecutive sinus cycles. Although the magnitude of this response varied between individuals-as reflected by the SD of RMSSD and ΔPP - its direction was consistent (Figures 3, 4).

This observation is physiologically plausible: the post-pacing increase in RMSSD and ΔPP is compatible with enhanced parasympathetic modulation. One possible explanation is transient hemodynamic change (e.g., cardiac output/arterial pressure) with baroreceptor engagement and increased vagal efferent activity (La Rovere et al., 1995); however, this mechanistic pathway was not directly measured in the present study.

HRV analyzed in Holter ECG monitoring has long been used as a method for assessing the function of the autonomic nervous system, primarily reflecting its parasympathetic activity (Task Force of The European Society of Cardiology and The North American Society of Pacing and Electrophysiology Heart rate variability, 1996; Cygankiewicz and Zareba, 2013). The parameter assessed in this study - RMSSD between adjacent PP intervals - is an indica-tor of short-term variability dependent on parasympathetic tone. However, it has not previously been used to assess the short-term parasympathetic response to rapid pacing during EPS, nor calculated based on only 4 heart cycles. To date, the shortest, ultra-short periods in which RMSSD has been assessed were 60 s (Esco and Flatt, 2014), 30 s (Baek et al., 2015), and 10 s (Salahuddin et al., 2007), covering at least one respiratory cycle lasting about 10 s.

Until now, the above-mentioned HRV parameters have been used to assess parasympathetic tone based on long-term ECG recordings. Pachon et al. demonstrated the usefulness of HRV parameter analysis, including RMSSD, in evaluating the persistence of CNA effects. They showed a significant reduction in heart rate variability one and 2 years after CNA compared to Holter ECG recordings performed before CNA (Pachon et al., 2020).

Using HRV parameters for intraoperative assessment of parasympathetic tone - both under baseline conditions and after provocation with rapid AP - opens up new possibilities for real-time evaluation of changes in parasympathetic tone and for monitoring the effectiveness of pro-cedures that modulate the heart’s parasympathetic innervation. This applies both to procedures specifically aimed at parasympathetic neuromodulation - such as CNA - and to those in which parasympathetic neuromodulation is an unintended effect - for example, during pulmonary vein isolation or ablation of atypical atrial flutter.

It should be noted, however, that the analysis of only 4 PP cycles both before and after the rapid AP is particularly susceptible to errors resulting from atrial extrasystoles, artifacts, and ineffective atrial stimulation. In such cases, the measurement should be repeated at least 2 min after the previous stimulation.

This study did not assess the impact of atropine or cardioneuroablation on intraoperative HRV measurements, which is a limitation and requires evaluation in future studies. The disappearance of the described phenomenon of increased RMSSD and Δ PP after the administration of atropine or following cardioneuroablation would be conclusive evidence of the increase in parasympathetic tone induced by rapid AP as the cause of this phenomenon.

Future studies need to clarify whether the increase in RMSSD and ΔPP in response to rapid AP is not only qualitative but also quantitative and depends on the strength of the stimulus provided by rapid AP-dose-response relationship. Does it also correlate with both the frequency and duration of rapid AP.

Additionally, the impact of sedative and general anesthetic medications on the phenomenon described in this study should be evaluated in future research. Sedative and general anesthetic agents influence both the tone of the autonomic nervous system and can modulate its sensitivity to stimuli, including rapid AF. Ahmed et al. described an increase in the threshold for vagus nerve stimulation in response to general anesthetics in an animal model. This is particularly relevant in the context of using intraoperative HRV measurements as a tool to assess parasympathetic tone under different conditions: local anesthesia, analgosedation, or general anesthesia (Ahmed et al., 2021).

Even though classical autonomic tests based on reflex assessment - such as the Valsalva ma-neuver or deep breathing test - are considered the gold standard outside the operating room, their intraoperatively usage is limited, as they require strictly controlled environmental condi-tions and appropriate patient preparation. The operating room environment deviates significantly from these ideal conditions, and the reliability of results obtained intraoperatively has not yet been systematically evaluated.

Moreover, since ECVS is currently the standard for intraoperative control, performing auto-nomic tests to directly validate them against ECVS would be reasonable only after the full effect of anesthetic drugs has worn off - in practice, after the procedure is completed. Intraoperative HRV assessment does not have these limitations. It does not require patient cooperation. Repeated measurements of RMSSD and ΔPP under the same conditions could, in the future, allow for the evaluation of parasympathetic tone changes not only qualitatively, as with ECVS, but also quantitatively. However, before this happens, intraoperative HRV measurement should be validated under different anesthesia conditions and in conditions of parasympathetic neuromodulation using atropine challenge or after cardioneuroablation.

Accordingly, at this stage the post-pacing RMSSD/ΔPP change should be regarded as a physiological observation and a research signal rather than a standalone clinical endpoint.

## Limitations

- Short-term HRV analysis in this study was based on only 4 consecutive sinus cycles per measurement point, limiting the statistical reliability of RMSSD and ΔPP estimations. While such a brief measurement window may reflect immediate responses to pacing, it may also amplify physiological noise and reduce precision. Notably, the study did not standardize or monitor respiratory activity (e.g., rate, depth, or waveform), which may have affected the magnitude of respiratory sinus arrhythmia -related variability.
- Nevertheless, the observed consistency between resting timepoints (A vs. C) and the systematic increase in RMSSD after both stimulations (A→B and C→D), combined with low baseline bias and variability, argue against substantial confounding by spontaneous autonomic fluctuations. The data suggest that the pacing-induced changes were genuine and primarily attributable to the intervention.
- Although the intraclass correlation coefficient (ICC) and Lin’s concordance correlation coefficient (CCC) were also computed for A vs. C and B vs. D, both yielded low values (A vs. C: ICC = 0.39; CCC = 0.38, B vs. D: ICC = 0.24; CCC = 0.22), suggesting weak absolute agreement. These coefficients are known to be highly sensitive to low inter-subject variance and small sample sizes. Given that RMSSD and ΔPP were calculated from only 4 sinus cycles per period, ICC and CCC were not considered reliable in this specific context. Bland-Altman analysis and Spearman correlation, due to their robustness to such constraints, were therefore prioritized for interpretation.
- ΔPP was calculated as the difference between the longest and shortest sinus cycle within a 4-beat window, making it inherently sensitive to single outlier values. This approach can exaggerate or obscure physiological trends, particularly in small samples. Moreover, its statistical reproducibility was limited, as evidenced by the high standard deviations observed in Bland-Altman analysis. Importantly, ΔPP as defined in this study has not been formally validated as a standard metric of short-term HRV, and no established normative values or thresholds exist for clinical interpretation. Nevertheless, ΔPP retains practical utility due to its ease of manual calculation and intuitive interpretation. In settings where only brief rhythm segments are available - such as during electrophysiologic procedures - it may offer a rapid, if coarse, insight into momentary shifts in autonomic tone. This data supports the use this technique to validate only sinus node denervation.
- The analyses were based on short beat sequences, which increases susceptibility to measurement noise and annotation errors (e.g., imperfect PP delineation, or signal artifacts, measurement errors). Accordingly, a small number of observations were flagged as outliers on Bland-Altman plots and appeared as discordant trajectories on second-order difference plots (SODP). These outliers may reflect technical artifacts or true inter-individual variability, including paradoxical responses to rapid AP. Because SODP is descriptive and Bland-Altman is primarily intended to assess agreement rather than causality, these tools were used to highlight potentially problematic segments and to support cautious interpretation; the primary conclusions were based on the main between-period comparisons.

## Conclusions

- Rapid atrial pacing induces a response in the form of an increase in RMSSD and ΔPP, which most likely results from increased parasympathetic tone.
- The parasympathetic response to rapid atrial pacing can be measured intraoperatively by analyzing changes in HRV parameters: RMSSD and ΔPP.
- The parasympathetic response to rapid atrial pacing is repeatable and can be measured multiple times during the same procedure.
- Assessing the influence of the parasympathetic nervous system on heart rhythm in response to rapid atrial pacing, measured using RMSSD and ΔPP, could in the future be used to verify the parasympathetic impact after various electrophysiological procedures, such as pulmonary vein isolation or CNA. However, this method only describes its effect on the sinus node and does not provide any information about parasympathetic influence on the atrioventricular node and requires validation in further studies.

## Data Availability

The raw data supporting the conclusions of this article will be made available by the authors, without undue reservation.
